# Attitudes toward Others Depend upon Self and Other Causal Uncertainty

**DOI:** 10.1371/journal.pone.0087677

**Published:** 2014-02-04

**Authors:** Stephanie J. Tobin, Matylda M. Osika, Mia McLanders

**Affiliations:** 1 School of Psychology, University of Queensland, Brisbane, Queensland, Australia; 2 Department of Psychology, University of Houston, Houston, Texas, United States of America; Universidad de Granada, Spain

## Abstract

People who are high in causal uncertainty doubt their own ability to understand the causes of social events. In three studies, we examined the effects of target and perceiver causal uncertainty on attitudes toward the target. Target causal uncertainty was manipulated via responses on a causal uncertainty scale in Studies 1 and 2, and with a scenario in Study 3. In Studies 1 and 2, we found that participants liked the low causal uncertainty target more than the high causal uncertainty target. This preference was stronger for low relative to high causal uncertainty participants because high causal uncertainty participants held more uncertain ideals. In Study 3, we examined the value individuals place upon causal understanding (causal importance) as an additional moderator. We found that regardless of their own causal uncertainty level, participants who were high in causal importance liked the low causal uncertainty target more than the high causal uncertainty target. However, when participants were low in causal importance, low causal uncertainty perceivers showed no preference and high causal uncertainty perceivers preferred the high causal uncertainty target. These findings reveal that goal importance and ideals can influence how perceivers respond to causal uncertainty in others.

## Introduction

What is more desirable in another person: certainty or uncertainty? The answer likely depends on numerous factors, including the object of uncertainty, the perceiver’s goals, and the perceiver’s level of uncertainty. In the current research, we focused on *causal uncertainty*, or doubt about one’s own ability to understand the causes of social events [1]. Causal uncertainty is arguably one of the most toxic forms of uncertainty, as it undermines a person’s sense of prediction and control. As a result, people experiencing high levels of causal uncertainty are motivated to improve their understanding. In the current research, we examined how this motivation shapes attraction to high and low levels of causal uncertainty in others.

### Causal Uncertainty

Causal uncertainty has been primarily conceptualized and studied as an individual difference variable [1]. The term ‘causal uncertainty’ typically refers to beliefs about one’s attributional abilities. These beliefs are often accompanied by feelings of confusion and a motivation to improve one’s understanding. Causal uncertainty and the motivation to reduce it have implications for both psychological well-being and social perception. Researchers have found that higher levels of causal uncertainty are associated with higher levels of negative affect, neuroticism, depression, anxiety, and pessimism, and with lower levels of self-esteem and perceived control [1]–[3]. Longitudinal studies have further revealed that causal uncertainty both stems from and contributes to a lack of perceived control [4], [5]. The latter finding is consistent with Heider’s [6] classic assertion that understanding the causes of events is critical for a sense of prediction and control. Importantly, unless people are protected by the sense that they can either accept or adapt themselves to existing events, higher levels of causal uncertainty about one’s own outcomes lead to increases in negative affect over time [3]. Thus, causal uncertainty seems to have negative implications for psychological well-being.

According to the causal uncertainty model [7], [8], when one’s current level of causal uncertainty exceeds one’s desired level by a noticeable amount, people experience negative affect and a goal to reduce the discrepancy. After assessing the chances of successful uncertainty reduction, individuals may adopt an accuracy goal and pursue it by carefully gathering and processing relevant information. Research has consistently supported this idea, finding that higher levels of causal uncertainty are associated with selection of more diagnostic questions for an interview [9], greater use of individuating information [10] and situational constraints [11], greater attention to causal explanations [12], and greater correction of judgments for potential bias [13].

It is important to note that the careful processing observed among high causal uncertainty perceivers is thought to stem from a desire to reduce causal uncertainty and the accompanying negative affect, rather than from an inherent enjoyment of thinking, greater attributional complexity, or a positive orientation toward uncertain situations. Consistent with this idea, researchers have found that causal uncertainty is negatively correlated with the need for cognition, or the tendency to engage in and enjoy effortful thought [1]. Causal uncertainty is also negatively correlated with attributional complexity, or the possession of highly developed attributional knowledge structures (unpublished data). Lastly, causal uncertainty is uncorrelated with uncertainty orientation, or positive engagement with uncertain situations [14]. These correlations suggest that the thorough processing observed among high causal uncertainty perceivers in previous research was not due to an intrinsic enjoyment of thinking in uncertain situations or complex attributional knowledge, but rather, a desire to reduce uncertainty and negative affect.

From past research and theorizing, it seems reasonable to posit that people who are high in causal uncertainty would prefer a lower level of causal uncertainty, whereas those who are low in causal uncertainty would be satisfied with their level of causal understanding. In other words, although actual levels of causal uncertainty vary, there may be a common desire for low causal uncertainty. Considering actual and ideal levels of perceiver causal uncertainty helps us predict how perceivers might react to high and low levels of causal uncertainty in another person.

### Interpersonal Attraction

Research on interpersonal attraction has consistently revealed that people are attracted to similar others, including those who share their attitudes and attributes [15]–[17]. Exposure to similar others is thought to validate one’s own attitudes and attributes, producing a rewarding experience. That is, when another person shares one’s attitudes toward issues [15] or one’s reactions to anxiety-inducing stimuli (e.g., avoidance/repression vs. approach/sensitization [17]), people feel as though their own judgments and reactions are appropriate. Such pleasant validation experiences increase liking for the similar other.

Based on these findings, one might expect low causal uncertainty perceivers to prefer low causal uncertainty targets and high causal uncertainty perceivers to prefer high causal uncertainty targets. Indeed, such effects have been found with depression and social anxiety. Specifically, research has revealed that depressed individuals have more depressed friends and feel worse after interacting with a non-depressed person [18]. Other studies have found that socially anxious individuals choose friends who are socially anxious [19], and feel closer to socially anxious compared to non-anxious targets when discussing personal topics [20]. Thus, exposure to a high causal uncertainty target might provide a positive validation experience for a high causal uncertainty perceiver.

However, an alternate prediction emerges when we examine the literature on similarity to ideal selves and interpersonal attraction. This research has revealed that when a discrepancy exists between the actual and ideal self, people prefer targets that resemble their *ideal* self [21]–[23]. Exposure to targets that resemble the ideal self may be rewarding if perceivers experience vicarious fulfillment of their ideals or experience positive changes in reputation or attributes through their association with the target [23]. Alternatively, people may like targets that resemble the ideal self simply because they find the target’s attributes desirable and they like people with desirable attributes [22]. However, research has revealed that when a target *surpasses* the perceiver’s ideal self, the perceiver feels threatened and likes the target less as a result [21]. Indeed, when others outperform them in important domains, people tend to experience envy and derogate the high-performing other [24], [25].

Based on these findings, we predicted that low causal uncertainty perceivers would be most attracted to low causal uncertainty targets, as these targets resemble both their actual and ideal self. In contrast, high causal uncertainty perceivers would not necessarily prefer high causal uncertainty targets, because although such targets resemble their actual self, they may not reflect their ideal self. Instead, high causal uncertainty perceivers may show a slight preference for low causal uncertainty targets. Their desire for a lower level of causal uncertainty should draw them toward the low causal uncertainty target, but their high level of actual causal uncertainty should lead them to adopt a more uncertain ideal. This would help keep the goal of accurate understanding within reach [8].

### The Current Research

In the current research, we used two different approaches to manipulate target causal uncertainty. In Studies 1 and 2, we adopted Byrne’s direct approach which controls extraneous target characteristics and communicates a target’s attitudes or attributes directly, through responses on a questionnaire [15]–[17]. After measuring participants’ causal uncertainty levels, we showed them a causal uncertainty scale that had ostensibly been filled out by another person and that indicated a high or low level of causal uncertainty. In Study 3, we used a more naturalistic scenario that depicted an interaction between a target person and several other people. In all studies, attitude toward the target person was the main dependent variable. We predicted that participants would generally prefer low relative to high causal uncertainty targets, but that these preferences would be stronger among participants with low relative to high levels of causal uncertainty.

## Study 1

In Study 1, participants first completed the causal uncertainty scale themselves, then at least a week later, viewed a causal uncertainty scale that had ostensibly been filled out by another person. Depending upon condition, the responses indicated that the target person had either a moderate or extreme, high or low level of causal uncertainty. After viewing the target causal uncertainty scale, participants indicated their attitudes toward the target as well as the extent to which the target person resembled their ideal self. We predicted that participants would prefer low relative to high causal uncertainty targets, and that low relative to high causal uncertainty participants would have stronger preferences. We also thought that preferences for low relative to high causal uncertainty targets might become stronger as the level of target causal uncertainty goes from moderate to extreme. We examined similarity to the ideal self as a mediator.

### Method

#### Ethics statement

Ethics approval for Studies 1 and 2 was obtained from the University of Houston’s Committee for the Protection of Human Subjects. A written information sheet was provided to participants, but the committee waived the need for written informed consent from the participants as the research was low risk, some minor deception was necessary to test our hypotheses, participants’ rights and welfare were not adversely affected, and a full debriefing was provided at the end of the study.

#### Participants

One hundred sixty-seven undergraduate psychology students at a North American university were randomly assigned to view a target causal uncertainty scale that indicated a moderate or extreme, high or low level of causal uncertainty. The data from four participants were excluded because they were outliers (>3 SD from the mean) on one or more of the measures. After these exclusions, the sample consisted of 136 female and 27 male participants. Participants were mostly between the ages of 18–24 (82%) and ethnically diverse (30% Caucasian, 26% Hispanic/Latino, 20% Asian, 18% African American, and 6% other).

#### Procedure

Participants completed the causal uncertainty scale in an online questionnaire at least one week before they came into the lab. The causal uncertainty scale contains 14 items that refer to positive and negative outcomes that involve the self and others (e.g., “When I receive good grades, I usually do not understand why I did so well,” “When I see something bad happen to others, I often do not know why it happened”). Participants indicated the extent to which they agreed with each item on a 6-point scale (1 = strongly disagree, 6 = strongly agree). Higher scores indicate a higher level of causal uncertainty. Past research has demonstrated that the causal uncertainty scale has high internal consistency (Cronbach’s alphas ranging from.83 to.86) and adequate test-retest reliability over a 6-week period (*r*s ranging from.62 to.80, *p*s <.001 [1], [2], [4], [5]).

Based on descriptive statistics from a large dataset (n = 402, causal uncertainty scale *M* = 2.58, *SD* = 0.79), we created target causal uncertainty scale sheets that represented extremely low causal uncertainty (2 SD below the mean or a score of 1.00 on the causal uncertainty scale), moderately low causal uncertainty (1 SD below the mean or 1.79), moderately high causal uncertainty (1 SD above the mean or 3.36), and extremely high causal uncertainty (2 SD above the mean or 4.14). Response patterns were typical of those who scored in the range of interest. We filled in a hard copy of the scale and then scanned the sheet into the computer. Thus, participants viewed a digital copy of the target causal uncertainty scale. Participants were told that the researchers were investigating how people form impressions of others based on minimal information and that participants would view a questionnaire that another participant had filled out in an earlier wave of this research and then answer some questions about the person.

We assessed participants’ attitudes toward the target by asking them to rate on a 5-point scale (1 = not at all, 5 = extremely) the extent to which each of the following adjectives reflected their reactions to the person who supposedly filled out the questionnaire: likeable, favorable, and positive. Participants also rated on 7-point scales how similar and different the target was to the kind of person they would ideally like to be. Lastly, as a manipulation check, participants rated on 7-point scales how uncertain and confident the target seemed about why things happen.

### Results

The data files for all studies are available upon request from the first author (s.tobin@uq.edu.au). Prior to calculating scale scores, we reverse-scored appropriate items and examined Cronbach’s alpha. Then, we averaged participants’ responses to the causal uncertainty scale (α = .88) and created indices that tapped attitudes toward the target (α = .83), perceived target uncertainty (α = .82), and similarity to the ideal self (α = .83).

#### Manipulation check

We regressed perceived target causal uncertainty on target causal uncertainty level (−1 = low, +1 = high), target causal uncertainty extremity (−1 = moderate, +1 = extreme), centered participant causal uncertainty, and all interactions. This analysis yielded significant main effects of target causal uncertainty level, β = 0.55, *t*(155) = 8.29, *p*<.001, and target causal uncertainty extremity, β = 0.18, *t*(155) = 2.68, *p* = .008. As intended, the high relative to low causal uncertainty targets were seen as more uncertain. In addition, the extreme relative to moderate causal uncertainty targets were seen as more uncertain. The latter effect was unexpected, but the effect of extremity appeared to be somewhat greater when comparing the moderately high causal uncertainty target (*M* = 4.43, *SD* = 1.16) to the extremely high causal uncertainty target (*M* = 5.42, *SD* = 1.31) than when comparing the moderately low causal uncertainty target (*M* = 2.60, *SD* = 1.54) to the extremely low causal uncertainty target (*M* = 3.05, *SD* = 2.13).

#### Attitudes toward the target

**Figure 1:**
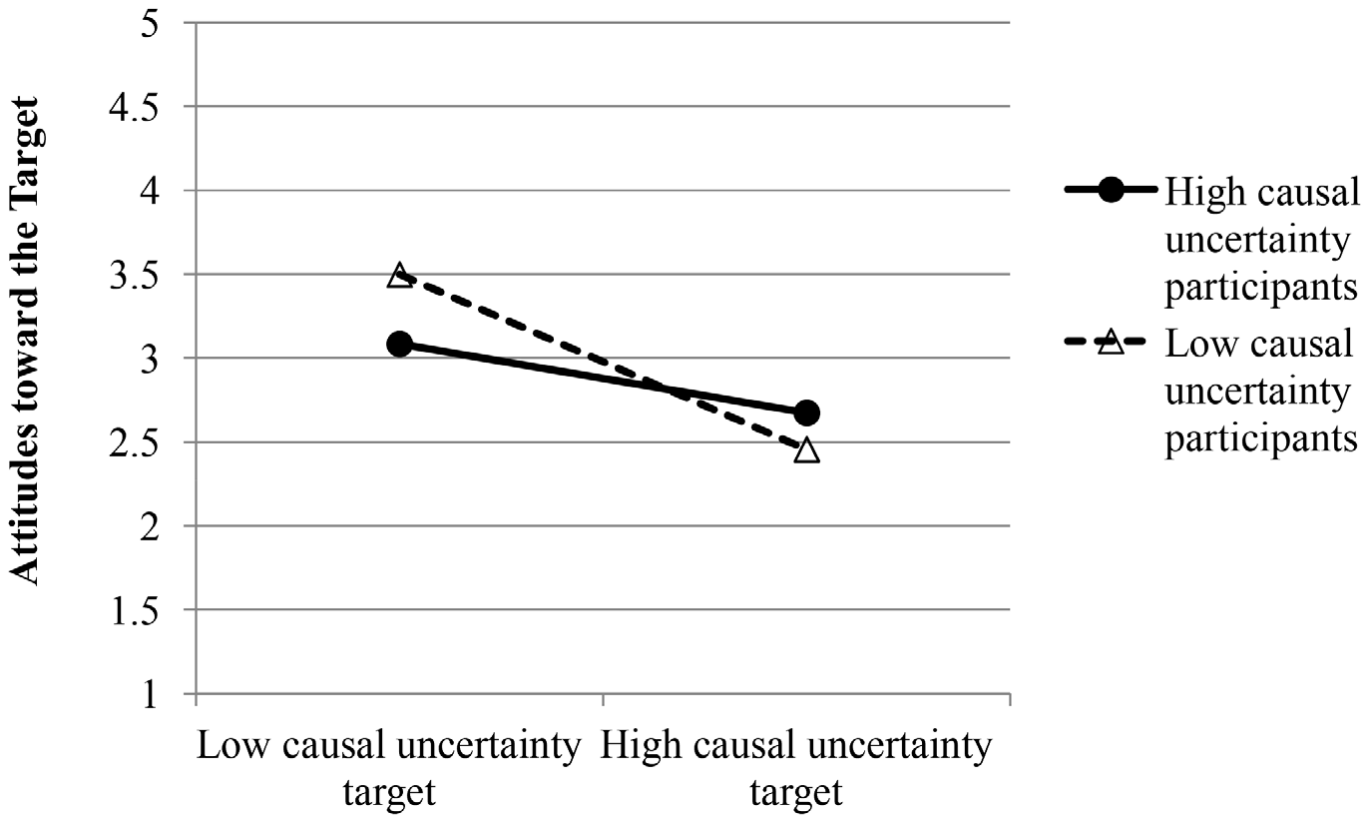
Attitudes toward the target as a function of participant and target causal uncertainty in Study 1.

To test our main hypothesis, we regressed attitudes toward the target on target causal uncertainty level, target causal uncertainty extremity, centered participant causal uncertainty, and all interactions. This analysis yielded significant main effects of target causal uncertainty level, β = −0.43, *t*(155) = −6.07, *p*<.001, and target causal uncertainty extremity, β = −0.19, *t*(155) = −2.66, *p* = .009. Participants indicated more positive attitudes toward low relative to high causal uncertainty targets, and toward moderate relative to extreme targets. The Target Causal Uncertainty Level X Participant Causal Uncertainty interaction was also significant, β = 0.18, *t*(155) = 2.59, *p* = .011. Simple slopes tests revealed a stronger preference for low relative to high causal uncertainty targets among low causal uncertainty participants (1 SD below the mean), β = −0.61, *t*(155) = −6.03, *p*<.001, than among high causal uncertainty participants (1 SD above the mean), β = −0.24, *t*(155) = −2.43, *p* = .016. See Figure 1.

#### Similarity to the ideal self

Next, we regressed similarity to the ideal self on target causal uncertainty level, target causal uncertainty extremity, centered participant causal uncertainty, and all interactions. This analysis yielded significant main effects of target causal uncertainty level, β = −0.41, *t*(155) = −5.82, *p*<.001, and target causal uncertainty extremity, β = −0.19, *t*(155) = −2.71, *p* = .008. Participants rated the low relative to high causal uncertainty targets and moderate relative to extreme targets as better resembling their ideal selves. The Target Causal Uncertainty Level X Participant Causal Uncertainty interaction was also significant, β = 0.19, *t*(155) = 2.68, *p* = .008. Simple slopes tests revealed a stronger effect of target causal uncertainty on similarity to the ideal self among low causal uncertainty participants, β = −0.60, *t*(155) = −5.93, *p*<.001, than among high causal uncertainty participants, β = −0.22, *t*(155) = −2.19, *p* = .030.

#### Mediational analysis

Previous analyses revealed significant Target Causal Uncertainty X Participant Causal Uncertainty interactions on attitudes toward the target and similarity to the ideal self. To examine whether similarity mediated the observed effects of target and participant causal uncertainty on attitudes toward the target, we conducted a mediational analysis using Hayes’ [26] PROCESS bootstrapping program (model 8). Attitude toward the target was the outcome variable, target causal uncertainty level was the predictor variable, similarity to the ideal self was the mediator, centered participant causal uncertainty was the moderator, and target causal uncertainty extremity was a covariate. The indirect effect of the Target Causal Uncertainty Level X Participant Causal Uncertainty interaction on attitudes toward the target through similarity to the ideal self was significant, effect = .11, SE = .05, 95% confidence interval [.02, .20]. An examination of the conditional indirect effects revealed that they were significant at both low (1 SD below the mean), effect = −.28, SE = .06, 95% confidence interval [−.41, −.17], and high (1 SD above the mean), effect = −.10, SE = .05, 95% confidence interval [−.21, −.02], levels of participant causal uncertainty. Additionally, the direct effect of target causal uncertainty on attitude toward the target remained significant for low causal uncertainty participants, effect = −.23, SE = .08, *t* = −2.93, *p* = .004, but not for high causal uncertainty participants, effect = −.10, SE = .07, *t* = −1.36, *p* = .18.

### Discussion

Study 1 revealed that participants liked the low causal uncertainty targets more than the high causal uncertainty targets, and that this preference was stronger among low causal uncertainty relative to high causal uncertainty participants. Participants also thought that the low causal uncertainty target better resembled their ideal self, and this was particularly true for low relative to high causal uncertainty participants. A mediational analysis revealed that the effect of target and participant causal uncertainty on attitudes was mediated by similarity to the ideal self. These findings are consistent with the idea that attraction is driven by similarity to the ideal self, rather than the actual self [21]–[23]. However, the weaker effects among high relative to low causal uncertainty participants suggest that high relative to low causal uncertainty individuals may indeed hold more uncertain ideals. We examined this possibility directly in Study 2.

The extremity of the target’s causal uncertainty had only main effects on attitudes and similarity to the ideal self. Extreme targets were seen as less similar to the ideal self and were liked less. This effect may be due to the fact that the extreme targets were seen as more uncertain than the moderate targets, even in the case of the low causal uncertainty targets. It is possible that the lack of variability in the extreme low causal uncertainty target’s responses (i.e., all 1’s) made the responses seem unrealistic, and raised doubts about the target’s understanding. We addressed this issue in Study 2 by introducing some variability into the extreme low causal uncertainty target’s responses.

## Study 2

We had two goals in Study 2. First, we sought to address alternative explanations by assessing constructs related to participant causal uncertainty and target causal uncertainty. Second, we examined causal importance and ideal causal uncertainty as predictors of attitudes toward the target. To simplify the design, we dropped extremity as a factor in Study 2 and focused only on extremely high and low levels of target causal uncertainty.

To rule out alternatives, we assessed several constructs that were related to participant causal uncertainty to see if they were responsible for any of the observed effects of participant causal uncertainty. Specifically, we assessed participants’ levels of self-esteem, depression, and trait affect. We did not expect these constructs to account for the predicted effects of participant causal uncertainty. We also had participants rate the target on dimensions related to causal uncertainty (i.e., self-esteem, depression) and those that might go along with extremely low causal uncertainty (i.e., conceitedness) to see if inferences about these constructs might be driving the observed effects of target causal uncertainty. We did not expect these constructs to account for the predicted effects of target causal uncertainty. Lastly, we examined perceptions of the accuracy of the target’s understanding so that we could rule out the possibility that high causal uncertainty participants doubted the accuracy of the low causal uncertainty target’s understanding.

To further test our proposition that high causal uncertainty participants hold more uncertain ideals, we assessed ideal causal uncertainty directly and examined it as a predictor of attitudes toward the target. We also assessed causal importance, or the value that individuals place on causal understanding. Causal importance has been found to magnify the effects of causal uncertainty [8], [12], [27]. For example, Tobin and Weary [12] found that high causal uncertainty participants thought carefully about causal persuasive arguments only if they were also high in causal importance. In the current research, we thought that causal importance might increase high causal uncertainty participants’ liking of the low causal uncertainty target, as high causal importance could orient participants toward self-improvement.

### Method

#### Participants

Ninety-eight undergraduate psychology students at a North American university were randomly assigned to view either a low or a high causal uncertainty target. Data from 11 participants were excluded: 4 participants did not complete the ideal causal uncertainty scale, 3 participants spent too little time on the initial set of questions (84 questions in under 4 minutes) or the target causal uncertainty scale (less than 1 second) and 4 participants were outliers (>3 SD from the mean) on one or more variables. After these exclusions, the sample consisted of 75 female and 12 male participants; 85% were between the ages of 18–24; 29.9% were Caucasian, 25.3% were Hispanic, 20.7% were Asian, 19.5% were African American, and 4.6% selected “Other”.

#### Procedure

Participants completed a number of questionnaires online. Among these were the causal uncertainty scale and a modified version of the causal uncertainty scale that asked participants to report their ideal level of agreement with each statement. The ideal causal uncertainty scale always came last. The other scales were the causal importance scale [12], the Rosenberg Self-Esteem Scale (RSES), the Center for Epidemiological Studies Depression Scale (CES-D), and the Positive and Negative Affect Schedule (PANAS). The order of these questionnaires was randomized. Participants then signed up for a lab session that took place at least one week later.

As in Study 1, participants completed the original causal uncertainty scale [1]. We will refer to the construct assessed by this scale as “actual causal uncertainty” to distinguish it from our new measure of ideal causal uncertainty.

To assess participants’ ideal level of causal uncertainty, we changed the instructions and response scale of the original causal uncertainty scale. Participants were asked to respond to the items based on how confident they would ideally like to be (1 = Ideally, I would like to “Strongly Disagree”, 6 = Ideally, I would like to “Strongly Agree”).

Six items assessed the importance that participants placed on causal understanding [12]. Participants rated the extent to which they agreed with items such as “I feel like it is important to be able to determine the actual cause or causes of events in my life” (1 = strongly agree, 6 = strongly disagree). Tobin and Weary [12] found that the causal importance scale had high internal consistency (α = .86) and adequate test-retest reliability over a 7-week period (*r* = .63, *p*<.001). The reverse-scored item was not included in the causal importance index in Study 2 or 3, as it reduced the reliability of the scale.

The RSES [28] contains 10 items that indicate positive (e.g., “I take a positive attitude toward myself”) and negative (e.g., “I certainly feel useless at times”) evaluations of the self. Participants rated the extent to which they agreed with each item on a 5-point scale (1 = strongly disagree, 5 = strongly agree). Higher scores indicate higher self-esteem.

The CES-D [29] contains 20 items that tap various symptoms of depression (e.g., “I felt sad,” “I felt that everything I did was an effort”). Participants rated how often they had felt each way during the past week on a 4-point scale [0 = rarely or none of the time (less than 1 day), 3 = most or all of the time (5–7 days)]. Higher scores indicate a higher level of depression.

The PANAS [30] contains 10 positive (e.g., excited) and 10 negative (e.g., upset) affective states. Participants rated the extent to which they generally felt each way on a 5-point scale (1 = very slightly or not at all, 5 = extremely).

Depending upon condition, participants viewed either a low (*M* = 1.14) or high (*M* = 4.14) causal uncertainty target sheet. The stimuli were identical to the extremely low and extremely high causal uncertainty target sheets used in Study 2 with one exception: we changed two of the extremely low causal uncertainty target’s responses from 1′s to 2′s to make the responses seem more realistic.

As in Study 1, three items assessed attitudes toward the target and two items assessed perceived target uncertainty. We also assessed perceived accuracy of the target’s understanding by asking participants to rate on 7-point scales how accurate they thought the target’s ideas were about why things happen and how well they thought the target understood why things happen. We assessed characteristics that might be related to target causal uncertainty by asking how conceited the target seemed, how depressed participants thought the target was, and how high they thought the target’s self-esteem was.

### Results

We first created indices that reflected actual causal uncertainty (α = .88), ideal causal uncertainty (α = .90), causal importance (α = .84), participant self-esteem (α = .90), participant depression (α = .90), trait positive affect (α = .92), trait negative affect (α = .87), attitudes toward the target (α = .81), perceived target uncertainty (α = .81), perceived accuracy of the target’s understanding (α = .89), and perceived target depression/self-esteem (α = .86). We examined perceived target conceitedness as a single item because when it was included in the depression/self-esteem index, it lowered the alpha to.66. A Cronbach’s alpha of at least.70 is generally desirable [31].

Actual and ideal causal uncertainty were positively correlated, *r = *.55, *p*<.001, indicating that higher levels of actual causal uncertainty were associated with more uncertain ideals. However, causal importance was not correlated with either actual causal uncertainty, *r* = −.04, *p* = .719, or ideal causal uncertainty, *r* = −.04, *p* = .716. Additionally, actual causal uncertainty and causal importance did not interact to predict ideal causal uncertainty, β = −0.08, *t*(83) = −0.79, *p* = .431.

#### Manipulation check

We regressed perceived target causal uncertainty on target causal uncertainty level (−1 = low, +1 = high), centered participant causal uncertainty (actual), centered participant causal importance, and all interactions. This analysis yielded only a significant main effect of target causal uncertainty level, β = 0.79, *t*(79) = 11.03, *p*<.001. As intended, the high causal uncertainty target (*M* = 5.69, *SD* = 1.21) was seen as more uncertain than the low causal uncertainty target (*M* = 2.46, *SD* = 1.45).

Similarly, when we examined the perceived accuracy of the target’s understanding, we found only a main effect of target causal uncertainty, β = −.56, *t*(79) = −5.79, *p*<.001. Participants thought that the low causal uncertainty target had a better understanding than the high causal uncertainty target.

#### Attitudes toward the target

**Figure 2:**
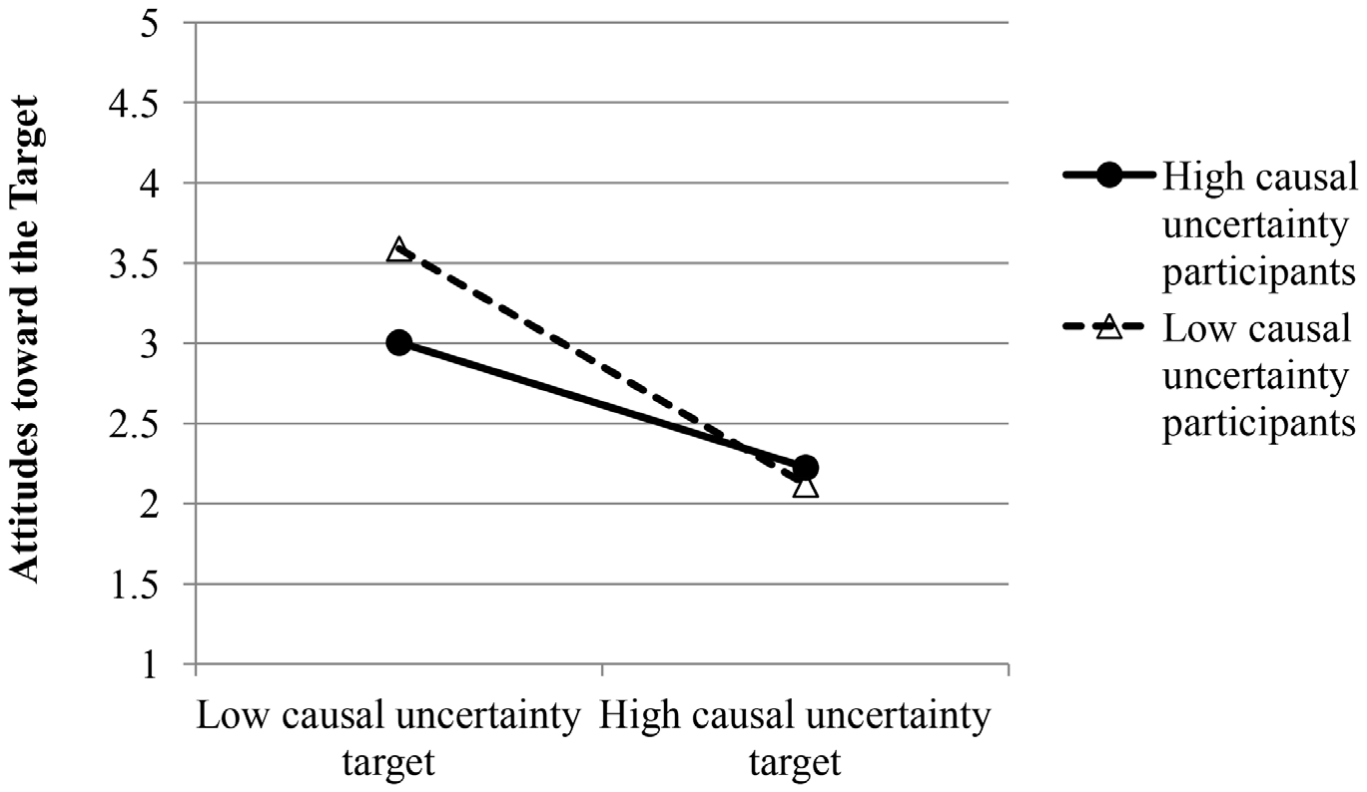
Attitudes toward the target as a function of participant and target causal uncertainty in Study 2.

Next, we regressed attitudes toward the target on target causal uncertainty, participant causal uncertainty (actual), causal importance, and all interactions. This analysis revealed significant main effects of target causal uncertainty, β = −.63, *t*(79) = −7.23, *p*<.001, and participant causal importance, β = .19, *t*(79) = 2.04, *p* = .045. Attitudes were more positive toward the low relative to high causal uncertainty target and among high relative to low causal importance participants. The Target Causal Uncertainty X Participant Causal Uncertainty interaction was also significant, β = .19, *t*(79) = 2.05, *p* = .044. See Figure 2. Simple slopes tests revealed that both high, β = −.44, *t*(79) = −3.57, *p* = .001, and low, β = −.82, *t*(79) = −6.13, *p*<.001, causal uncertainty participants liked the low causal uncertainty target better than the high causal uncertainty target. However, the magnitude of the effect was larger among low compared to high causal uncertainty participants.

We repeated the above analysis with ideal participant causal uncertainty in place of actual participant causal uncertainty. This analysis revealed a significant main effect of target causal uncertainty, β = −.63, *t*(79) = −7.16, *p*<.001, and a significant Ideal Participant Causal Uncertainty X Target Causal Uncertainty interaction, β = .18, *t*(79) = 2.08, *p* = .041. Although participants with high, β = −.45, *t*(79) = −3.63, *p*<.001, and low, β = −.81, *t*(79) = −6.45, *p*<.001, causal uncertainty ideals both liked the low causal uncertainty target better than the high causal uncertainty target, the magnitude of the effect was larger among participants with lower causal uncertainty ideals. Thus, the effects of ideal participant causal uncertainty mirrored those of actual participant causal uncertainty.

Next, we examined whether any of the individual difference variables related to participant causal uncertainty might account for the effects of participant causal uncertainty. Higher levels of actual participant causal uncertainty were associated with higher levels of negative affect, *r* = .42, *p*<.001, and depression, *r = *.49, *p*<.001, and lower levels of self-esteem, *r* = −.49, *p*<.001. In separate regressions, we regressed attitudes toward the target on one of the related individual difference variables, target causal uncertainty, and the interaction term. There were no significant effects involving depression, self-esteem, positive affect, or negative affect, *p*s >.20.

#### Inferences about the target

Lastly, we examined the other target ratings as a function of actual participant causal uncertainty, participant causal importance, target causal uncertainty, and all interactions. We observed only a target causal uncertainty main effect on perceived depression/self-esteem, β = .63, *t*(79) = 6.82, *p*<.001. Participants thought the high causal uncertainty target was more depressed than the low causal uncertainty target. When we examined conceitedness, we observed a main effect of target causal uncertainty, β = −.28, *t*(79) = −2.70, *p* = .008, and a Target Causal Uncertainty X Participant Causal Uncertainty interaction, β = −.25, *t*(79) = −2.24, *p* = .028. Simple slopes analyses revealed that high causal uncertainty participants rated the low relative to high causal uncertainty target as more conceited, β = −.53, *t*(79) = −3.65, *p*<.001, but that low causal uncertainty participants did not, β = −.03, *t*(79) = −0.18, *p* = .862.

To examine whether the effects of participant causal uncertainty and target causal uncertainty on liking were due to inferences about the target’s other characteristics, we ran a final set of analyses. In two separate regressions, we regressed attitudes toward the target on target causal uncertainty, participant causal uncertainty, participant causal importance, target characteristic (depression/self-esteem or conceitedness), and all interactions. In both regressions, the Target Causal Uncertainty X Participant Causal Uncertainty interaction remained at least marginally significant, *p*s = .053 (controlling for depression/self-esteem) and.041 (controlling for conceitedness). The only effect of other target characteristic was a main effect of target depression/self-esteem, β = −.49, *t*(71) = −5.03, *p*<.001. Targets who were perceived as being more depressed were liked less.

### Discussion

As in Study 1, we found that participants liked the low causal uncertainty target more than the high causal uncertainty target, and that low relative to high causal uncertainty participants showed a stronger preference for the low causal uncertainty target. Analysis of actual and ideal causal uncertainty in Study 2 indicated that high causal uncertainty participants held a more uncertain ideal than did low causal uncertainty participants. These different ideals predicted their liking of the high and low causal uncertainty targets. Participants with more uncertain ideals showed a weaker preference for the low relative to the high causal uncertainty target.

In Study 2, we were able to rule out the possibility that high causal uncertainty perceivers doubted the accuracy of the low causal uncertainty target’s understanding of why things happen. Participants thought the low causal uncertainty target had a more accurate understanding of why things happen than the high causal uncertainty target, and participant causal uncertainty had no effect on perceived accuracy. It was also not the case that the observed effect of target and participant causal uncertainty on attitudes was due to inferences about the target’s level of depression/self-esteem or conceitedness. Although the low causal uncertainty target was perceived as less depressed and more conceited (among high causal uncertainty participants) than the high causal uncertainty target, these inferences did not account for the observed effects of target and participant causal uncertainty on attitudes. Lastly, participants’ levels of trait affect, depression, and self-esteem did not influence their attitudes toward the target.

We tested participant causal importance as a potential moderator of target and participant causal uncertainty on attitudes, but found only a main effect. Higher causal importance was associated with more positive attitudes toward both targets. It is possible that the direct relevance of the causal uncertainty items to high causal importance participants’ goals created a positive reaction that spilled over to the target. We examined causal importance in Study 3 as well to test the role it played with more naturalistic materials.

## Study 3

Our main goal in Study 3 was to convey target causal uncertainty through everyday behavior, rather than responses on the causal uncertainty scale. Accordingly, we created a scenario in which the main character expressed either a high or low level of uncertainty about the causes of events in her life. As in Study 2, attitudes toward the main character served as the dependent variable, and we examined causal uncertainty and causal importance as predictors. However, because the main character expressed uncertainty about self-relevant events, we used self causal uncertainty as a predictor rather than overall causal uncertainty scores. The causal uncertainty scale consists of two highly correlated factors: causal uncertainty about one’s own outcomes (self causal uncertainty) and causal uncertainty about other people’s outcomes (other causal uncertainty [2]). It is appropriate to compute either an overall scale score or subscale scores, depending upon the nature of the experimental stimuli and outcome variables [2], [3]. In Studies 1 and 2, participants viewed the target’s responses to self and other causal uncertainty items, so it was appropriate to use overall causal uncertainty scores as our predictor. However, in Study 3, self causal uncertainty was more relevant to the type of causal uncertainty expressed by the target.

We expected to replicate the findings observed in Studies 1 and 2, such that we would find a preference for the low relative to the high causal uncertainty target and that this preference would be stronger among low relative to high causal uncertainty participants. We also examined causal importance as a moderator. Causal importance had only a main effect on attitudes toward the target in Study 2, such that higher causal importance levels were associated with more favorable attitudes toward all targets, rather than just the low causal uncertainty target. However, given that target causal uncertainty was conveyed in an interpersonal context in Study 3, we thought it might have a different effect. The interpersonal nature of the scenario might make the apparent benefit of association with low causal uncertainty individuals more apparent. Past research has found that people can capitalize on the strengths of those who resemble their ideals in ongoing relationships [32]. High causal uncertainty perceivers who value the goal of causal understanding (high causal importance) might realize that spending time with a low causal uncertainty other could help them improve their understanding. Low causal uncertainty perceivers who value causal understanding might also prefer to spend time with those who understand, although the self-improvement motive would be weaker for this group. Overall, then, we expected high causal importance to be associated with greater attraction toward the low causal uncertainty target.

### Method

#### Ethics statement

Ethics approval for Study 3 was obtained from the School of Psychology’s Ethics Review panel at the University of Queensland. A written information sheet was provided to participants, but the committee waived the need for written informed consent from the participants as the research was low risk and anonymous.

#### Participants

Ninety-six undergraduate psychology students at an Australian university were randomly assigned to read about either a low or a high causal uncertainty target. Data from 4 participants were excluded because they were outliers (>3 SD from the mean) on one or more variables. After these exclusions, the sample consisted of 63 female and 29 male participants who ranged in age from 17–48 (*M* = 19.62, *SD* = 4.30).

#### Procedure

Participants completed the study online. They began by filling out the causal uncertainty scale [1], the causal importance scale [12], the RSES [28], the CES-D [29], and the PANAS [30], in randomized order, followed by a filler questionnaire. The filler questionnaire was a 10-item attitude survey [12]. As noted earlier, we focused on causal uncertainty about one’s own outcomes as our measure of causal uncertainty in Study 3 [2]. Next, participants were asked to read a scenario about a person named Sarah.

The scenario opened with Sarah and her friend Beth, two first year university students, talking about a situation that had just occurred. They were at a party and had just been talking to two men. After talking for about 10 minutes, the men said they had to leave to go to another party across town. However, a little while later Sarah noticed that the men were still there talking to some other women. Sarah discussed the possible reasons for their behavior with Beth. Beth changed the subject to a recent statistics exam. Another friend of theirs, Chelsea, joined them and Sarah brought up the situation with the men again.

In the high causal uncertainty version of the scenario, Sarah expressed uncertainty about the men’s behavior, wondering if they had been lying or if it was something she said. When Beth offered an alternative explanation, she remained uncertain and raised yet another possibility. When Beth changed the subject to the statistics exam, Sarah said she had received a high score, but did not understand why. Beth suggested it was due to her studying hard, but Sarah remained uncertain. Sarah brought up the incident with the men again at the end of the scenario after Chelsea had joined them, but remained uncertain about the reasons for their behavior and stated that she “just can’t figure people out sometimes.”.

In the low causal uncertainty version of the scenario, Sarah expressed confidence in a single explanation for the men’s behavior (they were “players” who had lied about needing to leave) and resisted an alternative explanation from Beth. When Beth changed the subject to the statistics exam, Sarah said she had received a high score and agreed when Beth suggested that she must have studied hard. When Chelsea joined them, Sarah brought up the men again, reiterated her original explanation, and claimed that she could always spot “players”.

As in Studies 1 and 2, we assessed attitudes toward the target, similarity to the ideal self, perceived target uncertainty, the perceived accuracy of the target’s understanding, and perceptions of the target’s level of conceitedness, depression, and self-esteem. The items were the same as those used in Studies 1 and 2, although we asked participants to rate “Sarah” rather than “the person who filled out the questionnaire.” The items were presented in a fixed order, in the order described. Lastly, to ensure that we had created a realistic scenario, we asked participants to rate how realistic the scenario was (1 = not at all realistic, 7 = very realistic) and how often they encounter people like Sarah in their everyday lives (1 = never, 7 = very often).

### Results

We first created indices that reflected participant causal uncertainty (α = .85), participant causal importance (α = .81), participant self-esteem (α = .90), participant depression (α = .91), trait positive affect (α = .90), trait negative affect (α = .88), attitudes toward the target (α = .79), perceived target uncertainty (α = .88), perceived accuracy of the target’s understanding (α = .75), and similarity to the ideal self (α = .79). The alpha for the perceived depression, self-esteem, and conceitedness items was only.34, and the highest alpha for any two items was.50. Accordingly, we examined the items individually rather than creating an index.

#### Manipulation check

We regressed perceived target causal uncertainty on target causal uncertainty level (−1 = low, +1 = high), centered participant causal uncertainty, centered participant causal importance, and all interactions. This analysis yielded only a significant main effect of target causal uncertainty, β = 0.85, *t*(84) = 14.15, *p*<.001. As intended, the high causal uncertainty target (*M* = 6.24, *SD* = 0.95) was seen as more uncertain than the low causal uncertainty target (*M* = 2.45, *SD* = 1.54).

We were primarily interested in the mean levels of perceived realism of the scenario and of Sarah. However, we did check to see whether these ratings were influenced by target causal uncertainty, participant causal uncertainty, participant causal importance, or any interactions between these variables. They were not, *p*s >.06. Overall, participants thought the scenario was quite realistic (*M* = 5.41, *SD* = 1.03) and reported encountering people like Sarah fairly often (*M* = 4.62, *SD* = 1.22).

Next, we examined the perceived accuracy of the target’s understanding. We observed a main effect of target causal uncertainty, β = −.44, *t*(84) = −4.73, *p*<.001. Participants thought that the low causal uncertainty target had a more accurate understanding than the high causal uncertainty target. We also observed Target Causal Uncertainty X Participant Causal Uncertainty, β = .27, *t*(84) = 2.80, *p* = .006, Target Causal Uncertainty X Participant Causal Importance, β = −.24, *t*(84) = −2.51, *p* = .014, and Participant Causal Uncertainty X Participant Causal Importance, β = .27, *t*(84) = 2.62, *p* = .010, interactions. Simple slopes analyses revealed that at high levels of participant causal importance (1 SD above the mean), low relative to high causal uncertainty targets were seen as having a more accurate understanding, β = −.68, *t*(84) = −5.00, *p*<.001, and high relative to low causal uncertainty participants thought the target had a more accurate understanding, β = .35, *t*(84) = 2.71, *p* = .008. However, at low levels of participant causal importance (1 SD below the mean), neither target causal uncertainty, β = −.20, *t*(84) = −1.53, *p* = .130, nor participant causal uncertainty, β = −.28, *t*(84) = −1.59, *p* = .115, predicted perceived accuracy. Furthermore, the effect of target causal uncertainty on perceived accuracy was significant at low levels of participant causal uncertainty, β = −.71, *t*(84) = −5.19, *p*<.001, but not at high levels of participant causal uncertainty, β = −.16, *t*(84) = −1.24, *p* = .219.

#### Attitudes toward the target

**Figure 3:**
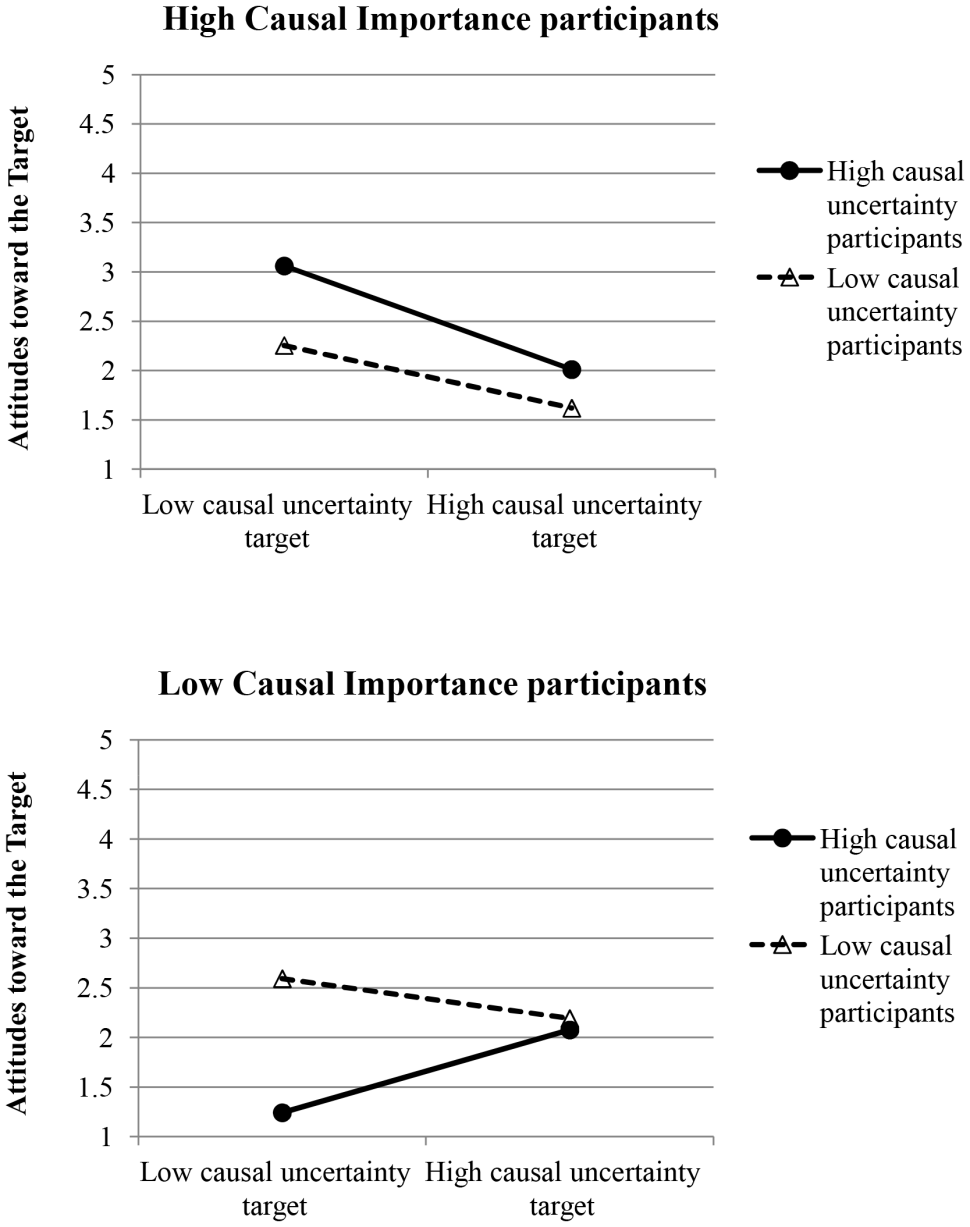
Attitudes toward the target as a function of participant causal uncertainty, participant causal importance, and target causal uncertainty in Study 3.

Next, we regressed attitudes toward the target on target causal uncertainty, participant causal uncertainty, participant causal importance, and all interactions. This analysis revealed a main effect of target causal uncertainty, β = −.23, *t*(84) = −2.45, *p* = .017, as well as Target Causal Uncertainty X Participant Causal Importance, β = −.39, *t*(84) = −4.01, *p*<.001, Participant Causal Uncertainty X Participant Causal Importance, β = .41, *t*(84) = 3.95, *p*<.001, and Target Causal Uncertainty X Participant Causal Uncertainty X Participant Causal Importance, β = −.25, *t*(84) = −2.45, *p* = .017, interactions. We conducted simple slopes analyses to examine the 3-way interaction; see Figure 3. These analyses revealed that participants who were high in causal importance had more favorable attitudes toward the low relative to high causal uncertainty target. This was true for high causal uncertainty/high causal importance participants, β = −.78, *t*(84) = −3.89, *p*<.001, and for low causal uncertainty/high causal importance participants, β = −.47, *t*(84) = −2.53, *p* = .013. However, when participants were high in causal uncertainty and *low* in causal importance, they had more favorable attitudes toward the high relative to low causal uncertainty target, β = .62, *t*(84) = 2.64, *p* = .010. Low causal uncertainty/low causal importance participants liked the two targets equally, β = −.30, *t*(84) = −1.37, *p* = .176.

As in Study 2, we examined whether any of the individual difference variables related to causal uncertainty might account for the effects of participant causal uncertainty on attitudes toward the target. Higher levels of participant causal uncertainty were associated with higher levels of negative affect, *r* = .40, *p*<.001, and depression, *r = *.40, *p*<.001, and lower levels of self-esteem, *r* = −.52, *p*<.001, and positive affect, *r* = −.34, *p* = .001. In separate regressions, we regressed attitudes toward the target on one of the related individual difference variables, target causal uncertainty, and the interaction term. There were no significant effects involving depression, self-esteem, positive affect, or negative affect, *p*s >.09.

#### Similarity to the ideal self

Next, we regressed similarity to the ideal self on target causal uncertainty, participant causal uncertainty, participant causal importance, and all interactions. This analysis yielded a main effect of target causal uncertainty, β = −0.26, *t*(84) = −2.49, *p* = .015, and Target Causal Uncertainty X Participant Causal Importance, β = −0.22, *t*(84) = −2.07, *p* = .042, and Participant Causal Uncertainty X Participant Causal Importance, β = 0.25, *t*(84) = 2.17, *p* = .033, interactions. Simple slopes analyses revealed that at high levels of participant causal importance, low relative to high causal uncertainty targets were seen as closer to the ideal self, β = −0.48, *t*(84) = −3.16, *p* = .002, and high relative to low causal uncertainty participants thought the target was more similar to their ideal, β = 0.36, *t*(84) = 2.48, *p* = .015. At low levels of participant causal importance, however, neither target causal uncertainty, β = −0.04, *t*(84) = −0.26, *p* = .796, nor participant causal uncertainty, β = −0.23, *t*(84) = −1.15, *p* = .255, predicted perceived similarity to the ideal self.

#### Mediational analysis

Previous analyses revealed a significant Target Causal Uncertainty X Participant Causal Uncertainty X Participant Causal Importance interaction on attitudes toward the target and a Target Causal Uncertainty X Participant Causal Importance interaction on similarity to the ideal self. To examine whether similarity mediated the observed effects of target and participant causal uncertainty on attitudes toward the target, we conducted mediational analyses using Hayes’ [26] PROCESS bootstrapping program (model 12). Attitude toward the target was the outcome variable, target causal uncertainty was the predictor variable, similarity to the ideal self was the mediator, and centered participant causal uncertainty and causal importance were moderators. Because none of Hayes’ available models fit our pattern of results exactly, we estimated the indirect effect of target causal uncertainty on attitudes toward the target via similarity to the ideal self at four combinations of participant causal uncertainty and causal importance.

The indirect effect was significant when participants were high (1 SD above the mean) in both causal uncertainty and causal importance, effect = −.17, SE = .08, 95% confidence interval [−.33, −.02]. However, the indirect effect was not significant at other combinations of causal uncertainty and causal importance; low causal uncertainty/high causal importance: effect = −.13, SE = .08, 95% confidence interval [−.27, .03]; high causal uncertainty/low causal importance: effect = −.01, SE = .09, 95% confidence interval [−.17, .19]; low causal uncertainty/low causal importance: effect = −.01, SE = .09, 95% confidence interval [−.20, .17].

The direct effect of target causal uncertainty on attitudes was significant among high causal uncertainty/high causal importance participants, effect = −.35, SE = .12, *t* = −2.88, *p* = .005, and among high causal uncertainty/low causal importance participants, effect = .43, SE = .14, *t* = 3.13, *p* = .002, but not among low causal uncertainty/high causal importance participants, effect = −.19, SE = .11, *t* = −1.73, *p* = .088, or among low causal uncertainty/low causal importance participants, effect = −.19, SE = .13, *t* = −1.49, *p* = .141.

Thus, it seems that with more naturalistic stimulus materials that portray high and low causal uncertainty targets in an interpersonal context, high causal uncertainty/high causal importance individuals prefer low to high causal uncertainty targets in part because of their greater similarity to the ideal self. High causal uncertainty/low causal importance participants prefer high to low causal uncertainty targets, but this effect was not mediated by similarity to the ideal self (i.e., the 95% confidence interval [−.17, .19] included zero). Although our earlier simple slopes analyses revealed that low causal uncertainty/high causal importance individuals preferred low to high causal uncertainty targets, neither the direct (p = .088) nor the indirect effect (95% confidence interval [−.27, .03]) was significant for this group with bootstrapping.

#### Inferences about the target

As in Study 2, we examined participants’ perceptions of the target’s level of depression, self-esteem, and conceitedness. We conducted a series of regressions in which we regressed target ratings on participant causal uncertainty, participant causal importance, target causal uncertainty, and all interactions. We observed main effects of target causal uncertainty on perceived depression, β = .32, *t*(84) = 3.14, *p* = .002, self-esteem, β = −.56, *t*(84) = −6.14, *p*<.001, and conceitedness, β = −.37, *t*(84) = −3.62, *p* = .001. The low relative to high causal uncertainty target was seen as less depressed, with higher levels of self-esteem and conceitedness. There was also a significant Participant Causal Uncertainty X Participant Causal Importance interaction on perceived depression, β = −.24, *t*(84) = −2.08, *p* = .040. However, none of the simple slopes were significant, *p*s >.06.

To examine whether the effects of participant causal uncertainty and target causal uncertainty on liking were due to inferences about the target’s other characteristics, we ran a final set of analyses. In three separate regressions, we regressed attitudes toward the target on target causal uncertainty, participant causal uncertainty, participant causal importance, target characteristic (depression, self-esteem, or conceitedness), and all interactions. In all regressions, the previously observed Target Causal Uncertainty X Participant Causal Uncertainty X Participant Causal Importance interaction became nonsignificant, *p*s ranged from.135 to.322, but the Target Causal Uncertainty X Participant Causal Importance interaction remained significant, *p*s <.01. In terms of the alternate constructs, we observed main effects of perceived depression, β = −.25, *t*(76) = −2.33, *p* = .022, self-esteem, β = .31, *t*(76) = 2.52, *p* = .014, and conceitedness, β = −.26, *t*(76) = −2.28, *p* = .025, and a Depression X Participant Causal Importance interaction, β = .22, *t*(76) = 2.14, *p* = .036.

Simple slopes analyses revealed that when perceived target depression was included as a predictor, at high levels of participant causal importance, low relative to high causal uncertainty targets were rated more favorably, β = −.67, *t*(76) = −4.44, *p*<.001, and perceived depression was not associated with attitudes, β = −.04, *t*(76) = −0.26, *p* = .793. Conversely, at low levels of participant causal importance, high relative to low causal uncertainty targets were rated more favorably, β = .28, *t*(76) = 1.99, *p* = .050, and perceived depression was negatively associated with attitudes, β = −.46, *t*(76) = −3.16, *p* = .002.

Additional simple slopes analyses revealed that when perceived target self-esteem was included as a predictor, at high levels of participant causal importance, low relative to high causal uncertainty targets were rated more favorably, β = −.43, *t*(76) = −2.49, *p* = .015. Conversely, at low levels of participant causal importance, high relative to low causal uncertainty targets were rated more favorably, β = .39, *t*(76) = 2.26, *p* = .027. When perceived target conceitedness was included as a predictor, at high levels of participant causal importance, low relative to high causal uncertainty targets were rated more favorably, β = −.72, *t*(76) = −4.28, *p*<.001, and at low levels of participant causal importance, there was no effect of target causal uncertainty on attitudes, β = .05, *t*(76) = 0.32, *p* = .750.

### Discussion

Recall that we had predicted that similar to Studies 1 and 2, we would observe a stronger preference for low relative to high causal uncertainty targets among low relative to high causal uncertainty participants. Furthermore, we expected causal importance to further moderate our effect such that higher causal importance should increase attraction to the low causal uncertainty target. Overall, these hypotheses were supported. Low causal uncertainty perceivers liked the low causal uncertainty target more than the high causal uncertainty target when they were high in causal importance, and showed no preference when they were low in causal importance. High causal uncertainty perceivers liked the low causal uncertainty target more than the high causal uncertainty target when they were high in causal importance, but showed the opposite effect when they were low in causal importance: they liked the low causal uncertainty target *less* than the high causal uncertainty target.

When we examined similarity to the ideal self, we found that high causal importance perceivers thought the low causal uncertainty target was closer to their ideal selves than the high causal uncertainty target. However, low causal importance perceivers thought the two targets resembled their ideal selves to an equal extent. Our mediational analysis showed that the indirect effect of target causal uncertainty on attitudes via similarity to the ideal was significant only for perceivers who were high in both causal uncertainty and causal importance, those who would have the highest motivation for self-improvement.

As in Study 2, we found no effect of perceiver depression, self-esteem, or trait affect on attitudes toward the target. We also found that similar to Study 2, the low causal uncertainty target was rated as having higher levels of accurate understanding, self-esteem, and conceitedness, and a lower level of depression. However, unlike Study 2, the effect of target causal uncertainty on perceived accuracy was observed only among perceivers who were high in causal importance or low in causal uncertainty. Although all perceivers picked up on the target’s uncertainty level, their inferences about the accuracy of the target’s understanding depended upon their own levels of causal uncertainty and causal importance. It may have been easier to question the target’s understanding of events in Study 3 since specific examples were given.

We also found that controlling for the target’s perceived level of depression, self-esteem, or conceitedness rendered the 3-way interaction nonsignificant. However, the 2-way interaction between target causal uncertainty and participant causal importance remained significant. High causal importance perceivers showed a consistent preference for the low causal uncertainty target over the high causal uncertainty target, and low causal importance perceivers showed either a preference for the high causal uncertainty target over the low causal uncertainty target (with depression or self-esteem as predictors), or no difference (with conceitedness as a predictor). It is possible that the addition of other predictors reduced our power and that the Target Causal Uncertainty X Participant Causal Importance interaction was simply more robust than the 3-way interaction. The additional inferences about the target did not interact with participant causal uncertainty or causal importance to predict attitudes toward the target, with one exception: low causal importance perceivers were more influenced by the target’s perceived level of depression than were high causal importance perceivers. Future research should continue to examine these related constructs, but at this point, we do not think they can account for our predicted effects.

## General Discussion

In three studies, we examined the effects of perceiver and target causal uncertainty on interpersonal attraction. In Studies 1 and 2, we found that perceivers liked low causal uncertainty targets more than high causal uncertainty targets, and that this preference was stronger among low causal uncertainty perceivers compared to high causal uncertainty perceivers. Additional analyses revealed that high causal uncertainty perceivers held more uncertain ideals than did low causal uncertainty perceivers, and this explained their weaker preference for the low relative to high causal uncertainty target. That is, there was a moderately large positive correlation between actual and ideal causal uncertainty in Study 2, and ideal causal uncertainty mirrored the effects of actual causal uncertainty when we examined it as a predictor. Additionally, perceived similarity of the target to the ideal self mediated the effects of participant causal uncertainty and target causal uncertainty on attitudes in Study 1.

These findings are consistent with previous research which found that in instances of a discrepancy between actual and ideal attributes, individuals prefer others who resemble their ideals [21]–[23]. Our findings go beyond past findings by examining individual differences in actual and ideal levels on a specific dimension rather than giving individuals false feedback on their actual and ideal selves on content-free dimensions [21], [23] or assessing a range of attributes [22]. By examining a specific dimension, we can better understand the dynamic interplay between actual and ideal levels of a particular attribute. For instance, it is noteworthy that high causal uncertainty perceivers hold more uncertain ideals. This could help to keep their goal of accurate understanding within reach. According to the causal uncertainty model, perceivers will only attempt to improve their understanding if they think there is some chance that they will succeed [8]. Striving for a somewhat lower level of uncertainty should seem more attainable than striving for absolute certainty.

We also found that causal importance, or the importance that individuals place on the goal of causal understanding influenced attraction. In Study 2, we observed only a main effect of causal importance, such that higher causal importance was associated with more favorable attitudes toward all targets. However, in Study 3, we found the specific pattern we predicted, with higher causal importance predicting increased attraction to the low causal uncertainty target. That is, for low causal uncertainty perceivers, we observed greater liking for the low relative to high causal uncertainty target when perceivers were high in causal importance, and no preference between the two targets when perceivers were low in causal importance. For high causal uncertainty perceivers, we observed greater liking for the low relative to high causal uncertainty target when perceivers were high in causal importance, and the opposite preference when perceivers were low in causal importance (i.e., greater liking of the high relative to low causal uncertainty target). For high causal uncertainty/high causal importance perceivers, similarity to the ideal self mediated the effect of target causal uncertainty on attitudes toward the target. We think this indicates that high causal uncertainty/high causal importance perceivers are motivated to improve their understanding and that the interpersonal nature of the scenario highlighted the potential benefits of association with others who possess superior qualities in this valued domain. Although superior targets can be threatening [21] and provoke envy [24], [25], they can also help one improve, especially in the context of an ongoing relationship [32].

### Strengths and Limitations

We were able to rule out a number of alternative explanations in the current research. Specifically, we examined whether individual differences related to perceiver causal uncertainty or inferences about the target might be driving the observed effects. In Studies 2 and 3, we showed that participants’ levels of depression, self-esteem, and trait affect had no effect on attitudes toward the target.

We also examined inferences about the *target’s* levels of depression, self-esteem, and conceitedness in Studies 2 and 3. As we had anticipated, participants did infer lower levels of depression and higher levels of self-esteem and conceitedness in the low relative to high causal uncertainty target. These related attributes were associated with participants’ attitudes toward the target. Higher levels of perceived depression were associated with more negative attitudes toward the target (for all participants in Study 2, and for low causal importance participants in Study 3). And in Study 3, lower levels of self-esteem and higher levels of conceitedness were associated with more negative attitudes. However, perceptions of the target’s level of depression, self-esteem, and conceitedness did not seem to account for the observed effects. In Study 2, our Target Causal Uncertainty X Participant Causal Uncertainty interaction on attitudes remained at least marginally significant when we controlled for the other perceived target attributes. In Study 3, although our Target Causal Uncertainty X Participant Causal Uncertainty X Participant Causal Importance interaction became nonsignificant, the Target Causal Uncertainty X Participant Causal Importance interaction remained significant. This 2-way interaction reflected high causal importance participants’ preference for the low relative to the high causal uncertainty target and low causal importance participants’ preference for the high relative to low causal uncertainty target (when controlling for depression or self-esteem) or indifference (when controlling for conceitedness). As we mentioned earlier, we think the nonsignificance of the 3-way interaction when controlling for related target attributes might have been due to the reduction of power that occurs when four variables and all possible interactions are included as predictors. Another possibility is that the 3-way interaction was simply not reliable.

Conversely, we were able to show that similarity to the ideal self provided a good explanation for the observed results. Perceived similarity of the target to the ideal self mediated the effects of target and participant causal uncertainty on attitudes toward the target in Study 1, and for high causal uncertainty/high causal importance participants in Study 3. Furthermore, participants’ level of ideal causal uncertainty moderated the effect of target causal uncertainty on target liking in Study 2.

One limitation of Studies 1 and 2 is low ecological validity. Participants would rarely have access to another person’s scores on a causal uncertainty scale. However, causal uncertainty was conveyed in a more naturalistic manner in Study 3. Indeed, participants rated the scenario as realistic and reported encountering people like Sarah fairly often in their everyday lives. The scenario also allowed us to convey causal uncertainty in an interpersonal context. The behaviors in the scenario should give researchers a better idea of what to look for and bring out in real life interactions to better understand the effects of causal uncertainty on actual interactions. Research that has examined the effects of actor and partner causal uncertainty in actual interactions has produced mixed results. Boucher and Jacobson [33] found that when previously unacquainted individuals have an interaction, a person’s level of causal uncertainty does not affect how he or she is rated by the interaction partner. However, Jacobson and her colleagues have found that higher causal uncertainty predicts greater rejection by roommates (unpublished data). It is possible that causal uncertainty only manifests itself in observable behavior when individuals feel comfortable and secure in an interaction or when their motivation to reduce causal uncertainty is aroused by the context (like in our scenario). The current research demonstrates the effects of target causal uncertainty on attraction when target causal uncertainty is apparent to perceivers. Understanding *when* causal uncertainty becomes apparent in an interaction and *how* it is communicated through various verbal and nonverbal behaviors is equally interesting and should be examined in future research.

### Implications

Our findings illustrate the importance of assessing ideals. Future studies examining the effects of depression and anxiety on interpersonal attraction should also assess perceiver ideals. This may help us understand whether previous findings [18]–[20] were due to depressed and anxious participants holding different ideals than nondepressed and nonanxious individuals, or whether perceivers were instead showing a preference for actual over ideal similarity. If it is the latter, then researchers could examine moderators of the basis of attraction. Perhaps when a strong affective state is elicited, preference for validation overrides improvement motives.

Our findings also have implications for people’s social lives and psychological well-being. The finding that high causal uncertainty can reduce liking means that people experiencing high levels of causal uncertainty may be at risk for interpersonal rejection. Such interpersonal rejection would make it more difficult for high causal uncertainty individuals to fulfill their social needs and could generate additional uncertainty.

To return to the question we opened the paper with, we can now conclude that certainty is desirable in others, at least when it comes to causal uncertainty. This likely reflects perceivers’ adaptive desire for an adequate grasp of cause-and-effect relationships. Because uncertain perceivers adopt more uncertain ideals, preferences for low causal uncertainty targets can be muted. However, higher levels of causal importance can enhance preferences for low relative to high causal uncertainty targets. It would be interesting to extend this research by studying optimal levels of certainty in other domains and across cultures. There are bound to be domains and contexts in which uncertainty is more desirable than certainty: for instance, when modest estimates of one’s abilities are valued or when actual knowledge is low. The methods used in the current research could easily be adapted to pursue such questions.
